# A physiological concept unmasking vascular salt sensitivity in man

**DOI:** 10.1007/s00424-012-1128-5

**Published:** 2012-06-29

**Authors:** Hans Oberleithner

**Affiliations:** Institute of Physiology II, University of Muenster, Robert-Koch-Strasse 27b, 48149 Muenster, Germany

**Keywords:** Epithelial sodium channel (ENaC), Amiloride, Hypertension, Endothelial glycocalyx, Salt provocation test

## Abstract

About one third of the population worldwide is supposed to be salt sensitive which is a major cause for arterial hypertension later in life. For preventive actions it is thus desirable to identify salt-sensitive individuals *before* the appearance of clinical symptoms. Recent observations suggest that the vascular endothelium consists of two salt-sensitive barriers in series, the glycocalyx that buffers sodium and the endothelial cell membrane that contains sodium channels. Glycocalyx sodium buffer capacity and sodium channel activity are conversely related to each other. For proof of concept, a so-called salt provocation test (SPT) was developed that should unmask vascular salt sensitivity in humans at virtually any age. Nineteen healthy subjects, ranging from 25 to 63 years of age, underwent two series of 1-h blood pressure measurements after acute ingestion of a salt cocktail with or without addition of a sodium channel blocker effective in vascular endothelium. Differential analysis of the changes in diastolic blood pressure (*net ∆DP*) identified 12 individuals (63 %) as being salt resistant (net ∆DP = −0.05 ± 0.62 mmHg) and seven individuals (37 %) as being salt sensitive (net ∆DP = +6.98 ± 0.75 mmHg). Vascular salt sensitivity was not related to the age of the study participants. It is concluded that the SPT could be useful for identifying vascular salt sensitivity in humans already early in life.

## Introduction

For many years the kidney was thought to be the major player due to its ability to regulate body sodium. The focus has been put on the function of the epithelial sodium channel (ENaC) localized in the renal collecting duct. The activity of this channel is under the control of aldosterone, the key hormone regulating salt and water balance and thus maintaining body volume and arterial blood pressure. Malfunction of ENaC, e.g. due to inherited or acquired overactivity, is accompanied by arterial hypertension [4, 10, 11].

It is estimated that at least a third of the population worldwide is salt sensitive [38], i.e. blood pressure increases in response to the daily ingestion of large amounts of sodium, a habit widespread in our societies [18, 29]. The underlying mechanism is still obscure although it appears likely that, among other parameters, renal ENaC activity, and hence sodium accumulation in the body, plays a role in this scenario [2, 6, 13]. With the discovery of ENaC in endothelial cells [36] and its relation to the actin cytoskeleton [12], attention was directed towards the vascular system. The paradigm shift that the ENaC regulating hormone aldosterone, besides the kidney, also acts on extrarenal tissues [21] led to new insights into the pathophysiology of hypertension and the use of diuretics in cardiovascular diseases [3, 19, 27]. By using nanotechniques it was shown that sodium ions *as such* can alter the mechanical properties of endothelial cells and thus control endothelial function [16, 23]. A closer look at the luminal surface of endothelial cells revealed that the endothelial glycocalyx (eGC), an anionic biopolymer covering the inner surface of blood vessels [28, 35, 37], participates in the mechanisms of sodium homeostasis. The eGC is able to transiently store preferentially sodium ions that are bound, osmotically “silent”, to the negative charges of proteoglycans [1, 30]. The eGC is supposed to serve as a shield against rapid cellular sodium uptake via the endothelial ENaCs and thus slows, to some extent, the flux of sodium ions from the blood into the interstitium [22]. The access of plasma sodium entering the endothelial cells via the ENaCs is facilitated when the eGC is compromised, e.g. by high sodium intake [25]. As a consequence, diuretics as the ENaC blocker amiloride or related drugs become relevant for interfering with the function of the endothelium. Therefore, it was tempting to conclude that challenging the vascular system with an acute sodium load in presence of a potent ENaC blocker should transiently change plasma sodium concentration and/or blood volume, possibly reflected by a small but measurable change in blood pressure. This hypothesis was tested in healthy individuals by the so-called salt provocation test (SPT).

## Methods

### Procedure underlying the SPT

**Fig. 1:**
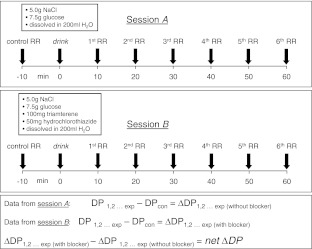
Experimental procedure and analysis of the blood pressure measurements. Sessions A and B were randomly performed with a time difference between sessions of at least 2 days

Nineteen healthy study participants (25–63 years of age) underwent two sessions of blood pressure measurements (upper arm, cuff method). At least 2 h prior to the experiments, study participants stopped any food intake. Measurements were performed in a sitting position. Figure 1 illustrates the procedure. In the beginning of the experiment, two control blood pressure measurements were performed before a salt cocktail was orally taken. The standard composition of this cocktail was 5.0 g NaCl and 7.5 g glucose, dissolved in 200 ml water. Then, in 10-min intervals, blood pressure was measured over 60 min. In session A the salt cocktail (no drugs added) was applied. In session B, at least 2 days later, drugs (100 mg triamterene and 50 mg hydrochlorothiazide) were added to the cocktail. It should be noted that the sequence of the sessions was randomly chosen (A, B or B, A), and the study participants were not biased by any working hypotheses prior to the individual sessions.

### Calculations

The analyses of the different diastolic blood pressure (DP) measurements are stepwise illustrated in Fig. 1. In short, ΔDP (experimental value minus control value) was calculated for each time point. Then, after completion of both sessions, the ΔDP obtained in absence of the drugs was subtracted from the corresponding ΔDP value obtained in the presence of the drugs. This result leads to a term called *net ΔDP*. It is used as a quantitative measure for the individual vascular salt sensitivity.

### Statistics

Data are shown as mean values ± SEM. Significance of differences was evaluated by the paired/unpaired Student’s *t* test if applicable. Overall significance level is *P* = 0.05 or less.

## Results

The baseline data of the study participants are summarized in Table 1. Study participants were healthy, ranging from 25 to 61 years of age, and were under no medical treatment.

**Table 1:** Baseline characteristics of study participants

Participant’s code number	Age (years)	BMI (body mass index)	Gender	Blood pressure before experiment (systolic/diastolic in mmHg)	Heart rate (bpm)
1	29	21.0	m	127/72	60
2	35	22.0	m	114/74	54
3	39	21.6	m	107/71	47
4	26	20.5	f	103/69	78
5	37	23.9	m	139/91	64
6	39	21.0	f	94/62	60
7	29	23.0	f	101/67	69
8	29	26.8	f	108/70	68
9	60	22.0	f	110/71	64
10	27	21.6	f	106/69	60
11	42	31.8	m	120/75	60
12	30	21.1	m	121/82	59
13	33	27.7	m	123/76	73
14	61	23.0	m	138/93	65
15	28	24.5	m	135/86	63
16	25	28.4	f	111/67	71
17	45	23.1	m	112/78	60
18	63	30.9	f	130/95	75
19	54	21.7	f	127/86	66

*m* male, *f* female

**Fig. 2:**
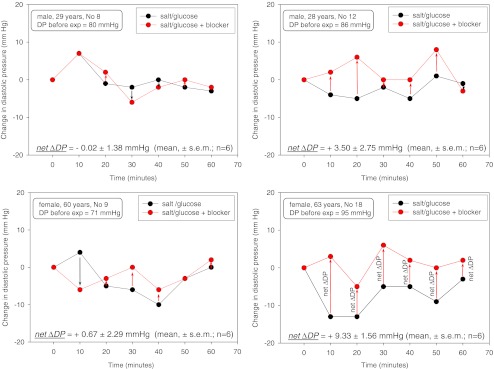
Changes of diastolic blood pressure, related to the corresponding control value (before the salt load), in response to salt load as indicated. Four different examples are shown. The *numbers in the insets* represent the study participant’s number and correspond to the numbers of Fig. 3

**Fig. 3:**
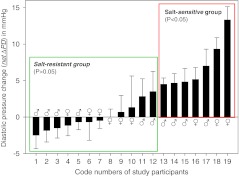
Net ΔDP (see Fig. 1 for definition) data of each study participant. The *green-framed left part* of the figure includes the 12 salt-resistant individuals, the *red-framed right part* includes the 7 salt-sensitive individuals

**Fig. 4:**
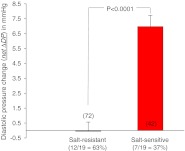
Mean values calculated from the sum of all ΔDP (see Fig. 1 for definition) obtained in the salt-resistant and the salt-sensitive participants (number of measurements indicated in/above the respective columns)

**Fig. 5:**
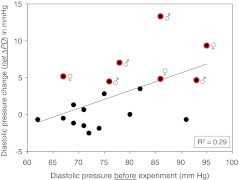
Relationship between the pre-experimental diastolic pressure and net ΔDP (see Fig. 1 for definition) of each individual. Data points from salt-sensitive individuals are marked with *red circles*. *R* ^2^ = correlation coefficient

**Fig. 6:**
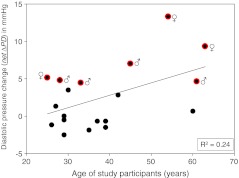
Relationship between participant’s age and net ΔDP (see Fig. 1 for definition) of each individual. Data points from salt-sensitive individuals are marked with *red circles*. *R* ^2^ = correlation coefficient

Figure 2 shows four examples of the two series of experiments. Diastolic pressure changes, related to the initial control diastolic pressure and measured over 60 min, are displayed. Diastolic pressure was preferred for the analysis since systolic pressure turned out to exhibit a considerable scatter during the experiment. Figure 2 (upper left part) shows the results of a 29-year-old male “non-responder”. The salt load, combined with diuretics, did not lead to a significant net ΔDP. Similar results were obtained in a 60-year-old female participant (lower left part). In the upper right part of Fig. 2, the results of a 28-year-old male study participant are displayed. The net ΔDP is “borderline” in terms of salt sensitivity though missing statistical significance (*P* > 0.05). Clearly salt sensitive is a 63-year-old female study participant whose net ΔDP is highly significant (right lower part, *P* < 0.001). The data are summarized in Fig. 3. Out of 19 study participants, the net ΔDP of 12 individuals was not statistically significant while 7 individuals showed a significant net ΔDP. Thus, the former group was termed salt resistant while the latter was termed salt sensitive. Figure 4 displays the two mean values of net ΔDP for the salt-resistant group (individuals with insignificant net ΔDP values) and the salt-sensitive group (individuals with significant net ΔDP values) based on the summarized blood pressure measurements (72 measurements in 12 salt-resistant individuals and 42 measurements in 7 salt-sensitive individuals). Figure 5 shows the correlation between the pre-experimental diastolic pressure of the study participants and net ΔDP. The data points of the salt-sensitive individuals are marked with red circles. Obviously, there is no significant correlation between the initial diastolic pressure (before the salt load) and the SPT score (net ΔDP values). Figure 6 shows the correlation between the age of the study participants and net ΔDP. Again, the data points of the salt-sensitive individuals were marked with red circles. Similar as in Fig. 5, there is no significant correlation between age of the study participants and the SPT score.

## Discussion

Salt and blood pressure in humans has been the subject of passionate debate over decades [5, 14, 17, 18, 34, 39]. Measurements of changes in blood pressure in response to pure saline infusion can be a possible way to predict salt sensitivity [31]. However, from a more mechanistic point of view, such a simple approach may be too error prone. The reason for this assumption is based on recent experimental evidence indicating a regulatory role of the endothelium in sodium metabolism. It was shown previously that an increase of plasma sodium concentration leads to the “stiff endothelial cell syndrome” [16] resulting in a decrease of nitric oxide release [23]. An increase in vascular smooth muscle tone is expected to occur, leading to elevated blood pressure. Thus, endothelial cells can function as “salt sensors” [24] and this function may decide on whether an individual is more or less salt sensitive. This view appears still too simplistic since the eGC has not yet been taken into account as a potentially relevant sodium regulatory system. The eGC, a negatively charged biopolymer, lines the inner surface of blood vessels and is able to buffer sodium to a significant amount [1, 25, 30]. Due to the buffering capacity of the eGC, the access for sodium entering the endothelial cells via sodium channels is limited. It has been estimated in in vitro experiments that about 10 % of the overall endothelial “sodium resistance” (i.e. the reciprocal of endothelial “sodium permeability”) is caused by an intact eGC [26]. When the eGC is poorly developed, then sodium ions have direct access to the endothelial plasma membrane. Then it depends on the abundance of ENaCs, regulated by aldosterone similar as in kidney, how avidly sodium can enter the endothelial cells [15, 25].

When we now reconsider the straightforward approach, namely using saline infusion to identify salt sensitivity [31], a problem is likely to occur: A vascular system with a well-developed eGC will readily buffer the infused sodium and thus prevent a clear-cut rise in blood pressure. On the other hand, a vascular system with a poorly developed eGC allows infused sodium to readily exit the vascular bed, mediated by the ENaCs. Again, a clear-cut rise of blood pressure in response to pure saline is unlikely.

A new perspective can be derived from the fact that eGC function and endothelial ENaC activity are conversely related to each other. This is indicated by the observations that a block of ENaC activity by amiloride is more efficient after enzymatic removal of the heparan sulphate residues from the eGC [15] or loss of the heparan sulphate residues caused by salt excess [25]. Taken together, a simple saline infusion protocol is not expected to clearly distinguish between salt-sensitive and salt-resistant individuals. It is obvious that vascular salt sensitivity depends on at least two parameters, namely on eGC function and ENaC activity.

**Fig. 7:**
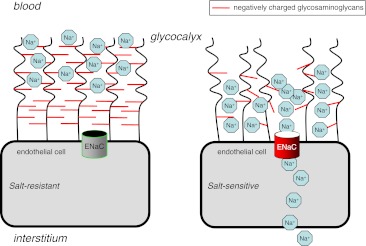
Schematic that shows the proposed mechanism. “Vascular salt resistance” is characterized by a well-developed glycocalyx with an optimum of negatively charged proteoglycans that buffer plasma sodium and thus impede the access of sodium to the respective channels of the endothelium. In contrast, vascular salt sensitivity is characterized by a poorly developed glycocalyx with a reduced number of negatively charged proteoglycans. Thus, plasma sodium has facilitated access to the sodium channels of the endothelium

This mechanistic view led us to design the SPT. Figure 7 illustrates this concept. The most adequate way, in our view, is to apply a salt load in the absence and in the presence of a sodium channel blocker. We assume that a salt-resistant vasculature (i.e. a well-developed eGC combined with a low ENaC abundance) should show no significant change in blood pressure when a salt load is combined with a sodium channel blocker. In this case, the ingested (or infused) sodium load should be buffered by the eGC and blood pressure should remain unaffected. However, in an individual with a salt-sensitive vasculature, the response is expected to be different. Ingested sodium is insufficiently buffered by the poorly developed eGC while the high activity of ENaCs found under these conditions is effectively inhibited by the sodium channel blocker. Therefore, ingested sodium circulates as “free (unbound) sodium” for some limited time in the vasculatory system thereby (transiently) increasing arterial blood pressure. The results of the present study show that 7 (out of 19) study participants responded with a significant SPT score (a positive net ΔDP). These participants (37 %) are supposed to be “salt sensitive”. The other individuals (63 %) did not respond and thus are supposed to be “salt resistant”.

We are aware that the number of study participants is small and thus limits conclusions concerning the percentage of salt-sensitive individuals in a certain population. However, a wide-scale clinical study was not the purpose of this approach. We rather tested a novel concept (proof of principle) of vascular salt sensitivity based on cellular mechanisms obtained recently in vitro [22].

The rationale behind applying glucose together with the salt load is to increase the velocity of sodium reabsorption in the intestinal tract. It is well documented that sodium is transported across the intestinal mucosa together with glucose in a molar stoichiometry of about 2:1 [7]. This stoichiometry is met by using the cocktail applied in the present study (5 g NaCl/7.5 g glucose).

Triamterene is used as a sodium channel blocker. This amiloride derivative is in common medical use [9]. A single dose of 100 mg is obviously sufficient to block the endothelial ENaC for a short period of time. A therapeutic application of amiloride as an ENaC inhibitor for the treatment of cardiovascular diseases has been proposed previously [33].

The combination of triamterene (100 mg) with hydrochlorothiazide (50 mg) has been chosen only for practical reasons because this is the mixture usually applied for medical purposes. Although some direct (rather vasodilatatory) effects of hydrochlorothiazides on the endothelium have been reported [8], the use of this diuretic is most likely irrelevant for identifying vascular salt sensitivity. However, this assumption should be further tested.

Addition of glucose to the salt cocktail will stimulate insulin secretion which is known to have some influence on nitric oxide release [20]. Thus, blood pressure may decrease slightly. Indeed we observed in a number of study participants a small transient decrease in blood pressure when the salt/glucose cocktail (without drugs) was ingested (compare Fig. 2, lower right part). This physiological response should not affect the impact of the SPT since it applies for both sessions (cocktail ± drugs) and thus cancels out. However, it explains that in another recent study where a salty soup without any addition of glucose was offered to humans, blood pressure slightly increased probably due to salt-induced volume expansion [32].

### Perspectives

Two 1-h sessions, at least 2 days apart from each other, could be sufficient to characterize the salt sensitivity of the vasculature in the human. This salt provocation test can be performed at virtually any age. It should help to predict the individual’s sensitivity for salt. The SPT is easily performed in general medical practice and may lead to preventive measures before cardiovascular malfunction occurs. Since “vascular salt resistance” of an individual obviously depends on a well-developed eGC, measures could be taken to improve eGC function.
